# Hydrofluoric Acid and Other Impurities in Toxic Perfluorooctane Batches

**DOI:** 10.1167/tvst.8.3.24

**Published:** 2019-05-29

**Authors:** Dirk-Henning Menz, Nicolas Feltgen, Thorsten Lechner, Helge Menz, Bernd-Kristof Müller, Joachim Dresp, Hans Hoerauf

**Affiliations:** 1 Pharmpur GmbH, Koenigsbrunn, Germany; 2 Department of Ophthalmology, University Medical Center, Goettingen, Germany; 3 Feldbergstr. 58a, Muenchen, Germany

**Keywords:** perfluorocarbon liquids, perfluorooctane, retinal toxicity, cytotoxicity, H-value, hydrofluoric acid, underfluorinated compounds, toxic impurities

## Abstract

**Purpose:**

The complications with cytotoxic perfluorooctane (PFO) batches reported in 2015 were attributed to reactive underfluorinated impurities whose chemical identity and behavior still need to be clarified.

**Material and Methods:**

We analyzed original packaged samples of Ala^®^octa batches involved in several reported cases of retinal toxicity. (A) The impurity profile was determined. (B) pH and fluoride ion content were measured. (C) Extraction with olive oil was performed to investigate differences in lipophilia among perfluorinated liquid (PFCL) as a measure for penetration of lipophilic cell membranes followed by measurements (A) and (B).

**Results:**

(A) The detected impurities can be divided into: (1) reactive underfluorinated compounds and their degradation products including hydrogen fluoride (HF), (2) nonreactive underfluorinated compounds, (3) surface active compounds, (4) nonreactive fluorinated compounds, and (5) leachables from primary packaging components. The highest acute toxic potential is associated with the impurities of group (1). (B) HF was detected as a degradation product of reactive underfluorinated impurities by relying on the pH values and fluoride ion content of the water extracts. (C) Lipophilic impurities dissolved in PFO migrate into lipophilic extraction medium. In particular, HF is rapidly transferred in this way.

**Conclusions:**

HF as degradation product of unstable or reactive underfluorinated contaminants seems of particular importance triggering the acute toxicity of affected PFO. Contamination related toxicity and unwanted side effects can only be reliably excluded via analytical controlled multistage, high-purification processes.

**Translational Relevance:**

In Ala^®^octa batches different impurities show retinal toxicity. HF seems of particular importance of the acute toxicity of PFO.

## Introduction

After decades using perfluorinated liquids (PFCL) successfully in vitreoretinal surgery, there have been repeated reports of retinal toxicity caused by individual batches and different manufacturers of these products since 2013. It is important to distinguish between acute toxicity and long-term tolerability. The reported symptoms describe acute toxic reactions that differ completely from the known side effects and tissue changes during long-term ophthalmological perfluorooctane (PFO) use.^1^,^2^

Serious toxic complications reported in 2015 after the intraocular use of PFO-batches produced by the Alamedics company (Dornstadt, Germany) were investigated and documented intensively.^3^ Previous work has shown that the toxic complications are due to reactive underfluorinated impurities,^4^ proven by determining the H-value, which quantifies such impurities and can be used as safety measure. The H-value is a quality parameter for fluorinated compounds in which, ideally, all hydrogen atoms have been replaced by fluorine atoms (PFCL). It indicates the concentration of the defective remaining hydrogen atoms. Its determination is carried out in two steps. Hydrogene fluoride (HF) is split off by a chemical transformation targeting precisely the residual protons. This is quantified potentiometrically in a subsequent analysis step. It should be performed as a limit test. The acceptance criterion should be “practically free from,” that means less than or equal to the detection limit (10 ppm).

The H-value was originally developed as a quality criterion for PFCL as blood substitutes^5^ and was later also applied to ophthalmological application.^6^ Its clinical relevance has recently been demonstrated as a safety parameter for PFO/PFCL endotamponades.^4^ The retinal toxicity of hydrogen-containing PFCLs was reported by Sparrow at al.^7^ and Chang et al.^8^ independent of the use of the H-value to characterize underfluorinated impurities. The given limit of 0.1 ppm was adopted to provide information on the raw material's manufacturer without disclosing their tests and validation. Their demand of the use of products with the highest possible quality is equivalent to the fixing of the acceptance criterion to the detection limit of suitable and validated procedures.

The H-value limit of 10 ppm was exceeded by several orders of magnitude in the PFO batches concerned. Individual batch differences in the H-value and associated cell growth inhibition were also striking.

The H-value used as the limit value of a sum parameter does not allow any conclusions about the specific identity of impurities. Since the toxic potential of the varying impurities is probably different, their characterization and the identity of the specific impurities are of high clinical interest.

Pastor et al.^3^ carried out the first investigations on the chemical constitution of the impurities contained in the PFO batches distributed by Alamedics and identified 1H,1H,7H dodecafluoro-1-heptanol, derivatives of perfluorooctanoic acid (PFOA), and ethylbenzene and p-xylene (1.4 dimethylbenzene)—known as leachables in pharmaceutical manufacturing. However, the relation between OH content and the leachables' content to the cytotoxicity observed in vitro remained vague. Due to large differences in the type and concentration of impurities in the affected batches, the need arose for a detailed quantitative and qualitative assessment of all impurities in order to provide a scientifically sound basis for general specifications. Also note that the impurities are dissolved in PFO and must first pass the interface to the tissue to be able to penetrate and interact with the ocular tissue and thus develop their toxic effect. Therefore, the purpose of our investigations focused on analyzing exactly those Alamedics PFO-batches (Ala^®^octa) involved in the reported cases and available in their original condition. Our analyses were carried out in three steps: (1) characterization of impurities using gas chromatography coupled with mass spectroscopy (GC/MS); (2) verification of the degradation of reactive underfluorinated impurities by testing HF content; and (3) extraction experiments to test in vivo the effect of conditions favoring tissue penetration on PFO impurity profiles.

## Material and Methods

### Material

Our investigations were performed on the original packaged samples of Ala^®^octa batches manufactured by Alamedics (listed in Table 1).

**Table 1:** Original Packaged Lots of Ala^®^octa, Manufactured by Alamedics GmbH&Co KG (Dornstadt, Germany) From Which Test Samples Were Collected

Ala^®^octa Lot #	Expiry Date
171214	2018-12
061014	2018-10
050514	2018-05
080714	2018-07
150414	2018-04
200114	2018-01
070714	2018-07
041213	2017-12

Presented tests in this paper were completed before the expiry date was reached.

We also tested the batches' raw materials: raw material R 1 = batch 1205052; raw material R2 = batch 1225572; R2/2 means: container 2 of raw material 2.

A commercial PFO, batch (PFO 33/15), with an H value < 10 ppm, manufactured and highly purified by Pharmpur GmbH (Koenigsbrunn, Germany), served as reference material.

Purified water according to Ph. Eur. 0008 was used as the extraction agent to extract water-soluble components.

To investigate the interaction with a purely lipophilic medium, olive oil (Fluka-Honeywell Spec. Chem., Seelze, Germany, 75343-1L, Lot: BCBM9115V) was used as an extracting agent.

To prepare external standard solution for quantifying detected impurities, the following materials were used:

- Dimethylbenzene mixture of isomers (VWR International GmbH, Darmstadt, Germany, 28975.291, Lot: 17K204015)
- 1H-PFO (Apollo Scientific Ltd., Manchester, United Kingdom, PC6140B, Lot: AS421860)
- Methyl-perfluorooctanoate (Alfa Aesar, Thermo Fisher Scientific, Karlsruhe, Germany, B23856, Lot: FA014458)
- Methyl-perfluorononanoate (Alfa Aesar, L16810, Lot: 90011213)
- 1H,1H,7H dodecafluoro-1-heptanol (Alfa Aesar, B20144, Lot: 10173880)
- Perfluoro-2-n-butyltetrahydrofurane (ABCR GmbH, Karlsruhe, Germany, AB103602, Lot: 1216897)
- PFOA (Sigma-Aldrich Chemie GmbH, Munich, Germany, 171468-5G, Lot: MKCC8766)

### Methods

#### (A) GC/MS

Equipment used includes the following: Shimadzu GC2010/QP2010Plus; column 30 m × 0.25 mm: Phenomenex, Zebron 5%-Phenyl-Aryl-95%-Dimethylpolysiloxane-Phase (1.0 μm).

The qualitative identification of the impurities was carried out through a library spectrum comparison (NIST 21/107).

In addition, impurity groups were identified by extracting the mass traces of selected key fragments of individual impurity types (key fragment CF_3_ with mass 69 for fluorinated impurities, key fragment COOCH_3_ with mass 59 for perfluoroalkanoic acid methylesters (methyl-perfluoroalkanoate), key fragment CF_2_CFO with mass 97 for oxygen-containing fluorinated impurities, key fragment C_3_F_3_ with mass 93 for fluorinated alkenes, key fragment CFCOH with mass 61 for fluorinated alcohols and key fragment C_6_H_5_CH_2_ with mass 91 for aromatic compounds (leachables).

Our semiquantitative results after testing impurities relied on evaluating the intensities detected of the related chromatographic peaks and expressed as relative intensities in arbitrary units or in case of the 1H-perfluoroalkane analogues, using the response factor and external standard of 1H-PFO; results are given in ppm.

The quantitative determination was carried out using the method of external standards (1,2 dimethylbenzene, 1,3 dimethylbenzene, 1,4 dimethylbenzene, ethylbenzene, Perfluoro-2-n-butyltetrahydrofurane, methyl-perfluorooctanoate, methyl-perfluorononanoate, PFOA, 1H,1H,7H dodecafluoro-1-heptanol and 1H-PFO).

#### (B) Verification of Degradation of Reactive Underfluorinated Impurities

The conversion of reactive underfluorinated impurities can be forced according to the general reaction scheme below.^4^

**Figure:**
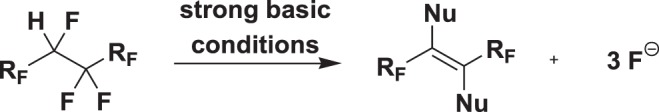

Equation 1: Reaction scheme of the general reaction of underfluorinated impurities with strong bases (RF = fully fluorinated substituent; Nu = nucleophiles that promote fluoride-cleavage under strong and harsh basic conditions; F^−^ = fluoride ions).

Unstable variants of underfluorinated impurities also transform spontaneously according to this equation:

**Figure:**
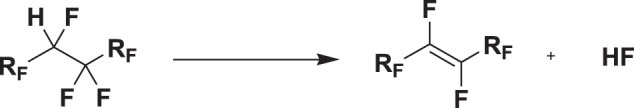

Equation 2: Reaction scheme of spontaneous conversion of unstable reactive underfluorinated impurities.

The HF formation promotes such reactive changes due to its high enthalpy of formation (−271 kJ/mol). The HF molecule can serve as a marker for both reaction types. For the HF detection, it must be extracted from the PFO with purified water in the first step in the volume ratio 10 (PFO) to 1 (water) in order to detect it later in the aqueous phase by determining the pH change (qualitative measure) and determining the fluoride content using fluoride-sensitive electrodes (quantitative measure).

The pH value was determined according to Ph.Eur. 2.2.3, USP <791>.

The fluoride content was determined according to the method described in detail earlier.^4^

Pastor et al.^3^ identified perfluoroalkanoic acid derivatives as impurities via GC/MS and suggested their free form via Raman spectroscopy investigations. To consider their contribution to pH changes, Fourier-Transform-Infrared-Spectrometer (FT-IR) studies were performed to determine the perfuorooctanoic acid content with 1-mm thick liquid cells, potassium bromide (KBr) windows, 64 scans, resolution 2 cm^−1^, using the peak at 1773 cm^−1^ and external standard method.

Equipment used includes the following: FT-IR Nicolet 380, Thermo Fisher Scientific, Inc. (Waltham, MA).

#### (C) Extraction Experiments

Extraction was carried out at a volume ratio of 1:1 at room temperature. After the phase separation, the investigations according to (A) and/or (B) were performed.

## Results

### (A) Identification of the Impurity Profiles

**Figure 1:**
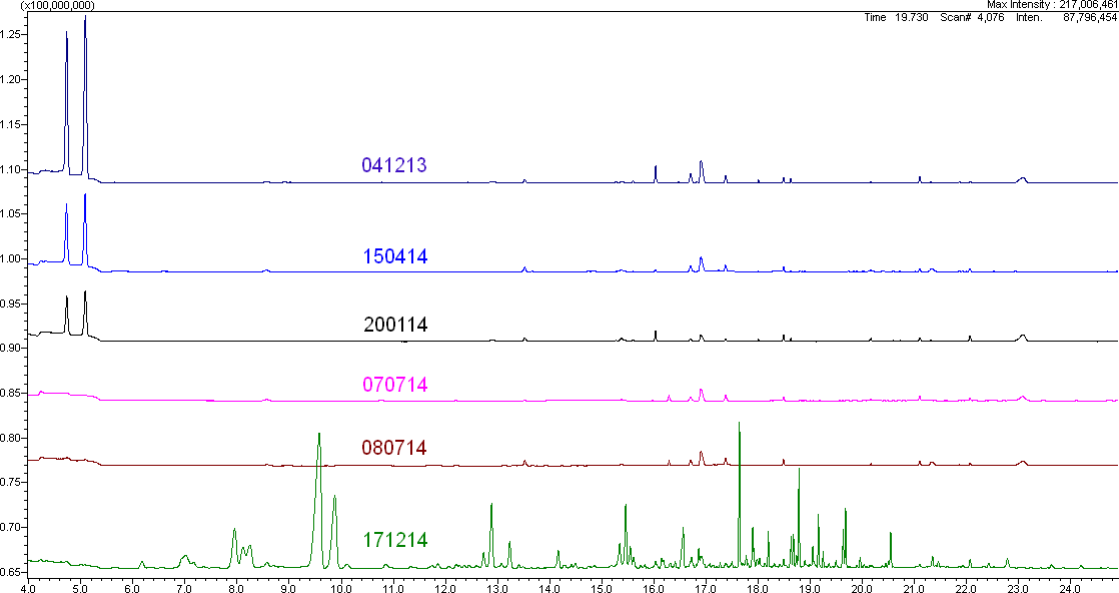
Impurity profiles of Ala^®^octa batches determined by GC/MS. (Batch 171214 is equivalent to 061014; 050514 is equivalent to 080714.)

The eight Ala^®^octa batches examined exhibited significantly different impurity profiles. Representative GC/MS chromatograms are illustrated in Figure 1.

**Figure 2:**
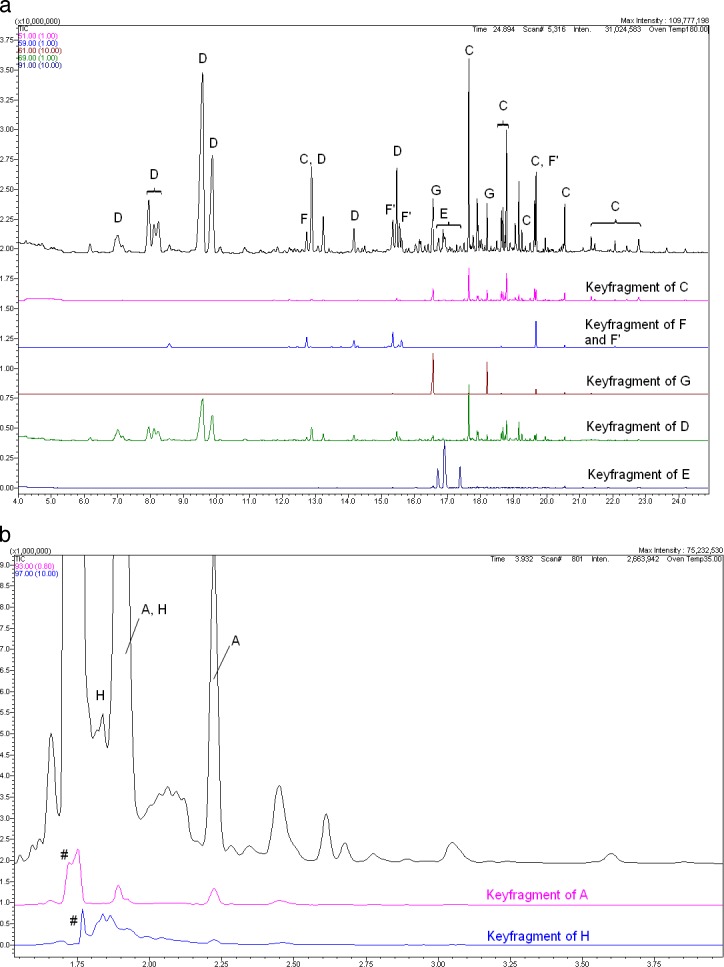
(a) Identification of impurities according to Table 2 using batch 171214; GC/MS results, retention time range over 4 minutes. (b) Identification of impurities according to Table 2 using batch 171214; GC/MS results, retention time range 1.5 to 3.8 minutes. # marks the artefact caused by the main component.

Figures 2a and 2b show our finding of the peak identification using batch 171214 as an example of batches with the highest number of detected impurities.

Three methods were used to identify the impurities:

- direct identification via library comparison of the mass spectra
- direct identification via reference substances
- indirect identification via key fragments, specific reactions, and plausibility considerations

The results are summarized in Tables 2 and 3 and are explained in greater detail below.

**Table 2:** Identification of Impurities via GC/MS Analysis Using the Data of Batch 171214

Peak	Identity of Impurities	Identification by GC MS Analysis			Plausible Source of Impurity
		Reference Material	Spectra Comparison ≥ 80% Conformance to NIST 21/107	Key Fragment m/z	
A	Perfluoro-alkenes	—	X	93	Degradation product
B	1H-PFO	X	X	51	By-product formed if reaction temperature is too low
C	1H-PF: Perfluoro alkane analogues	—	X	51	By-product formed if reaction temperature is too low
D	Perfluorinated compounds	—	X	69	By-products of fluorination
E	Ethylbenzene 1,4-, 1,3-, 1,2-dimethylbenzene (leachables)	X	X	91	rubber closure systems as contamination source
F	Methyl-perfluorooctanoate	X	X	59	Regional preferred raw material for easy access to n-PFO
F′	derivates of methyl-perfluoroalkanoate	—	X	59	
G	1H,1H,7H- dodecafluoro-1- heptanol	X	X	61	Regional preferred raw material for easy access to n-PFO
G′	1H,1H, fluoroalkan-1-ol	—	X	61	
H	Perfluorofurane	X		97	Cross contaminant during manufacturing

**Table 3:** Overview of the Impurity Profile of the Ala^®^octa Batches

Batch Number	Cell Growth Inhibition %^2^	Raw Material	Number of Cases Reported in Spain^1^	Reactive Underfluorinated Impurities (1)		Nonreactive Underfluorinated Impurities (2)		
						Containing Oxygen	Surface Active Impurities (3)	
				Underfluorinated Impurities Detected by Specific Reaction (Qualitative)	Perfluorinated Alkenes in Arbitrary Units (Semiquantitative)	Perfluorofurane in ppm (Quantitative)	1H- Perfluoroalkane- Analog in ppm (Semiquantitative)	1H-PFO in ppm (Quantitative)
171214	96	R 2/2		XXX	0.2	0.7	45	89
061014	94	R 2/2	8	XXX	0.2	0.7	66	59
050514	50	R 2/1	12	X	0.3	0.9	n.d.	89
080714	48	R2/1,2	3	X	1.0	2.6	540	115
150414	40	R 2/1	49	X	0.2	0.7	120	75
200114	39	R 1	3	X	1.0	3.0	590	875
070714	33	R 2/1		X	0.2	0.6	120	26
041213	23	R 1		X	0.2	0.8	110	292

X, minimum 1 species; XX, minimum 3 species; XXX, minimum 5 species; n.d., not determined; * Using 1000 ppm standard. Note: nonlinear below 100 ppm and interferences with other impurity traces.

**Table 3:** Extended

Batch Number	Reactive Underfluorinated Impurities (1), Surface Active Impurities (3), Containing Oxygen					Leachables (5)	Fully Fluorinated By-Products (4)
	Semiquantitative in Arbitrary Units		Quantitative				
	Methyl- Perfluoroalkanoate RT: 8.6 min	Methyl- Perfluoroalkanoate RT 14.3 min	PFOA Methyl ester in ppm	1H,1H,7H- dodecafluoro- 1-heptanol in ppm	PFOA Absolute in ppm (relative to 171214 in %)*	Sum of the Mix of the Dimethylbenzene Isomers and Ethylbenzene (ratio 82%:18%) in ppm (Quantitative)	Qualitative
171214	1.0	1.0	9	49	700 (100)	5	XXX
061014	0.8	0.5	5	25	—	—	XXX
050514	0.4	—	—	—	60 (9)	5	XXX
080714	0.4	—	—	—	50 (7)	30	XXX
150414	0.4	—	—	—	60 (9)	10	XXX
200114	—	—	—	—	105 (15)	20	XXX
070714	0.4	—	—	—	55 (8)	5	XXX
041213	0.2	—	—	—	60 (9)	15	XXX

#### Identification by Comparing the Registered Mass Spectra With Library Spectra (NIST 21/107)

The GC/MS method described in (A), optimized for analyzing trace impurities in PFCL, is highly sensitive and, therefore, enables the identification of considerably more impurities compared with earlier analyses^3^ (see Table 2). These impurities are highlighted in Figure 2. We also detected the impurities Pastor et al.^3^ identified but only in batches 171214 and 061014 (not shown).

In the case of perfluoroalkanoic acid derivatives, note that three peaks with different retention times were assigned to that compound type. This is due to the fact that these are three representatives of the same compound class whose mass spectra all share the same characteristic fragments (key fragments). Only one of them was identifiable when relying on reference standards (methyl-perfluorooctanoate). Traces of PFOA itself are not detectable with GC/MS.

#### Identification of Impurities by Comparison With Reference Substances

The comparison of the impurities' mass spectra gave us an initial indication of their identity. However, reliable results can only be derived if the spectra match is > 90% and contamination by these impurities is plausible. In any case, direct comparison to reference substances is more powerful than a pure library comparison, especially because quantitative assessments are additionally enabled.

Accordingly, for 1H-PFO, Perfluoro-2-n-butyltetrahydrofurane, ethylbenzene, dimethylbenzene, methyl-perfluorooctanoate, PFOA, and 1H,1H,7H dodecafluoro-1-heptanol we proved their contamination burden in the PFO samples by comparison with appropriate reference substances. Their concentrations were determined via an external standard method. Those findings are summarized in Table 3. FT-IR measurements had to be used for the corresponding PFOA analysis. In addition to the absolute values, their relative ratios were also provided to enable a straightforward comparison with the semiquantitative values stated by Pastor et al.^3^ We confirmed that the majority of the batches investigated contained very similar PFOA levels of despite varying degrees of cell growth inhibition. No obvious dose-dependence was detected.

#### Indirect Identification of Impurities via Mass Spectra Analysis

By extracting key fragments from the total mass spectra, impurities can be combined into groups.

This is shown in Figures 2a and 2b. The comparison of the total ion chromatogram with mass traces of key fragments enables the assignment of individual peaks, here to the fluorinated impurities with their key fragment CF_3_ (mass 69) and for the incompletely fluorinated impurities with the terminal group HCF_2_ (mass 51), derivatives of methyl-perfluoroalkanoates (mass 59) and 1H,1H fluoroalkan-1-oles (mass 61), leachables (mass 91), perfluoro-alkenes (mass 93), and perfluorofurane (mass 97).

#### Indirect Identification of Impurities by Specific Reactions

**Figure 3:**
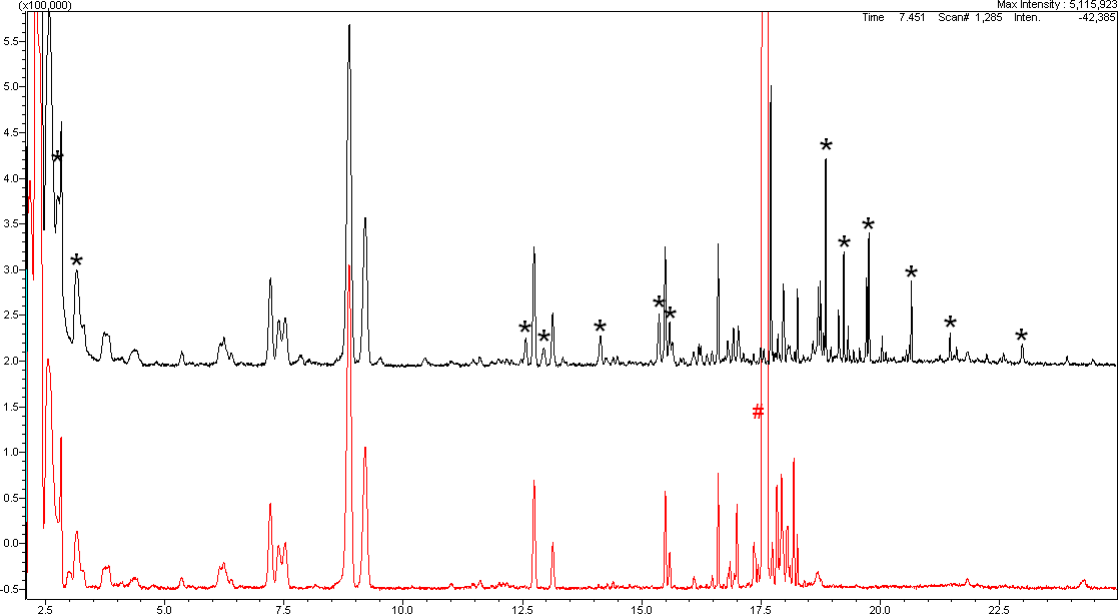
Comparison of the GC/MS results before and after reaction of batch 061014 with strong bases according to equation 1. The stars mark the reactive species in the initial state. # marks the solvent used for reaction (nonane).

Figure 3 illustrates our assignment of compounds to the group of reactive underfluorinated impurities. Identification is made by comparing the chromatograms before and after the chemical conversion of these impurities by reacting with strong bases, as required to measure the H-value (equation 1).^4^

#### Considering Plausibility

It should be noted that contamination of PFO batches by all the impurities listed in Table 2 can be attributed to the finished product's manufacturing process.

Ethylbenzene and dimethylbenzene are typical examples of leachables that can be released from the rubber material of stoppers and tip caps of primary containers (syringes or vials).

### (B) Verification of Degradation of Reactive Underfluorinated Impurities

Table 4 shows our pH measurements and fluoride content determination in the water extracts of the Ala^®^octa batches. HF as a marker of reactive underfluorinated impurities was detected by determining ionic fluoride (F^−^) in extracts and their pH values. Note that the free acid PFOA also contributes significantly to the pH value, but no free fluoride is formed by the extraction of the acid with water.

**Table 4:** Results of the pH Measurement and the F^−^- Content of Water Extracts of Ala^®^octa Batches

Alamedics Lot #	Cell Growth Inhibition in %^2^	H-Value in ppm^2^	Aqueous Extract	
			pH	F^−^-Content in mmol/L
171214	96	4500	2.08	2.3
061014	94	3000	2.27	n.d.
050514	50	2100	2.68	0.95
080714	48	2100	2.70	0.89
150414	40	1400	2.67	1.3
200114	39	2600	2.88	0.78
070714	33	1800	3.10	0.19
041213	23	3200	3.38	0.44
Pharmpur PFO 33/15	0	<10	5.85 (WFI)	0.01

WFI, water for injection.

### (C) Experiments to Extract Impurities With Olive Oil as a Model Substance to Simulate In Vivo Conditions of Tissue Penetration

Olive oil is a proven model substance to test differences in lipophilia, especially of PFCL.^9^

Comparison of the contamination profiles before and after extracting the PFO batches with olive oil reveals a reduction in impurities, indicating that PFO impurities, unlike PFO itself, can penetrate lipophilic aggregational phases such as tissue much better thanks to their being more lipophilic than PFO.

HF is a special kind of PFO impurity due to its chemical properties. It can dissolve in undissociated form in lipophilic media. Dissolution in aqueous media takes place under partial dissociation in H^+^ and F^−^.

Extracting via lipophilic media is much more effective in depleting HF than extracting via aqueous media. Total extraction via aqueous media requires several extraction steps, while extraction via olive oil is nearly complete after the first extraction (Table 5).

**Table 5:** Comparison of the Extraction Effectiveness Using Water or Olive Oil as Extraction Agent

Batch Number	Water Extract		Water Extract After Shaking With Olive Oil	
	pH	Fluorid mmol/L	pH	Fluorid mmol/L
Raw 2/2	2.33	2.16	3.71	0.01
171214	2.12	2.35	2.96	0.84

## Discussion

Our identification of the individual batch impurity profiles of the toxic Ala^®^octa batches confirms findings obtained by determining the H-values and contributes substantially to our understanding of the reported side effects; it also explains the varying degrees of toxicity of the tested batches.

The impurities can be classified in the groups below (see Table 3):

1. Reactive underfluorinated compounds and their degradation products including HF
2. Nonreactive underfluorinated compounds
3. Surface-active contaminants
4. Fully fluorinated byproducts
5. Leachables

### PFO Specific Impurities

#### 1. Reactive Underfluorinated Compounds and Their Degradation Products Including HF

This group is characterized by impurities that cannot be avoided in PFCL production. Depending on the effectiveness of the raw material's fluorination and the associated quality control, the individual impurity profile may both differ from batch to batch and change over time if unstable reactive impurities are present.

There is a dependency between the detected concentration of fluoride (F^−^, detected with fluoride sensitive electrodes) in the extracts and the cell growth inhibition induced by these batches. HF is dissolved in PFO undissociated as a lipophilic molecule. While the transition of HF from the PFO into the eye's aqueous environment is associated with dissociation and is thus limited by the dissociation equilibrium, its transition into lipophilic media (demonstrated here with olive oil) takes place rapidly and nearly completely already in a single extraction step. As a result, the small HF molecule can quickly penetrate tissue. This HF property has long been known, and it is the worst effect in case of skin contact.^8^ This may explain the severe acute intraocular tissue damage observed despite very short contact time during the surgery and which differs completely from the known side effects and tissue changes during long-term PFO use in ophthalmological applications.^1^,^2^ Although HF makes a major contribution to the batches' acute overall toxicity, the toxic potential of reactive underfluorinated impurities is the decisive risk factor of contaminated PFO. On the one hand, these compounds are a source for the resulting HF; on the other hand, they exert a toxic effect themselves.^10^

#### 2. Nonreactive Underfluorinated Compounds

This impurity group consists of compounds with a head-tail structure, such as the perfluorinated molecule chains that end in an HCF_2_ group. These compounds (called 1-H-perfluoroalkanes) are initial members of a homologous series of perfluoroalkylalkanes (also known in the literature as partially fluorinated compounds or FALK or RFRH.^11^–^13^).

They were developed as adhesion promoters and alternatives to PFCLs to stabilize emulsions of blood substitutes^14^ and as alternatives to PFCL in ophthalmology.^6^,^15^ Although not entirely fluorinated, these compounds are not reactive underfluorinated impurities.^6^,^16^ Terminal H-atoms in a fully fluorinated compound or CH_2_ groups strictly separated from a fully fluorinated part of the molecule are polarized to such an extent that bond strengthening occurs. Their chemical stability resembles that of perfluorocarbons, and they are harmless in regard to acute toxicity as long as the solvent character of the molecule's alkane part is overcompensated by the perfluorinated part and the contact time to the tissues are limited.^15^

1-H-perfluoroalkanes are typical by-products of PFCL synthesis when fluorination temperatures are too low.

#### 3. Surface-Active Contaminants

In addition to the compound groups contributing to overall toxicity, impurities with surface-modifying properties are a further risk factor.

They may belong to both the group of reactive underfluorinated compounds and the group of nonreactive underfluorinated compounds. An essential characteristic of this impurity group is its amphiphilic character, that is, the impurities interact with media of different polarity and hydrophilicity/lipophilicity. The surface activity may vary from surface-modifying action (1-H-perfluoroalkanes) to real surfactant behaviors (derivatives of perfluoroalkanoic acids).

The perfluoroalkanoic acid derivatives, including the acids themselves, play a special role. On the one hand, their effect as a strong surface-modifying substance must be taken into account and assigned accordingly to this group of impurities. On the other hand, they have a perfluorinated molecule part that can contain incompletely fluorinated positions in the fluorine chain due to the production process. Therefore, corresponding impurities must also be classified in the group of reactive, incompletely fluorinated impurities, and the reactions described above involving HF formation must be expected. In addition, in the case of free acids, the immediate corrosive action of the acid group must be taken into account. In contrast to HF, these acids must be classified as strong acids. The undesirable side effects of this class of substances are that they lower the interfacial tension against water, leading to an associated increase in interaction with tissue and enhanced penetration of lipophilic molecules; they also function as an adhesion promoter by interaction between the silicone oil and retinal surface (stickiness) during a subsequent silicone oil tamponade.^16^–^18^

While 1-H-perfluoroalkanes can form during synthesis of the raw material, the derivatives of perfluoroalkanoic acids are residues of unreacted starting compounds.

#### 4. Fully Fluorinated By-Products

For the sake of completeness, the fully fluorinated by-products of PFO production should also be mentioned; their chemical structure is so closely related to PFO and their properties so similar that they are difficult to separate from one another. This type of impurity is practically unavoidable in perfluorination reactions. Gervits stated in the 1990s that such by-products pose no risk to medical applications.^10^

### Non-PFO-Specific Impurities

#### 5. Leachables

The Ala^®^octa batches we investigated were additionally contaminated by leachables in different amounts because they had been filled into primary containers with different rubber closure materials. These can cause side effects, but this impurity group is not considered a PFO-specific contamination.

Leachables increase the risk of emulsification during subsequent silicone oil tamponade, as these lipophilic impurities accumulate in silicone oil.^18^,^19^ However, acute toxic reactions as described in affected batches are extremely unlikely to occur in the detected concentration range. The limit values for solvent residues in pharmaceuticals specified for dimethylbenzenes in the International Council for Harmonisation of Technical Requirements for Pharmaceuticals for Human Use guidelines Q3(R6)^20^ are undercut by a factor of 100 (permitted daily exposure) or 1000 (concentration maximum). This is accompanied by the fact that we found no correlation between the concentration of leachables and cell growth inhibition (Tables 3 and 4).

### Contribution to Acute Retinal Toxicity

Due to their different chemical composition and content (see Tables 3 and 4), the risks and side effects of the contamination groups differ. This is particularly relevant for their toxic potential. The reactive underfluorinated compounds (1) cause acute cytotoxic reactions due to their own toxic effect (especially when they contain double bonds) and the formation of HF already in the ppm range. An additional particular hazard potential lies in the fact that they can show both immediate and delayed (latent, depot) effects, depending on their reactivity.

Surface-active contaminants (3) are able to alter the barrier function of cell membranes and lead to long-term damage. They contribute only indirectly to acute toxicity by intensifying the interaction with other noxae. Heteroatom-containing compounds to which oxygen-containing impurities belong lead to response by macrophages and enhanced dispersion.^7^

The acute toxic reactions from affected batches described above require a fast-acting noxious agent at a high enough concentration.

In this context, the corrosive effect of the impurities presents as acids (HF, PFOA) must be particularly considered. The concentrations found in the individual batches are different, and the measured pH values are due to the contribution of both acids. Although the corrosive effect or dose-dependent damage to tissue^21^ by PFOA should not be underrated, the PFOA concentrations detected in individual batches fail to explain the varying cell growth inhibition these batches induced. This can only be explained by an additional effect.

However, since no significant difference was reported in the course and extent of the damage in different batches, the triggering noxious agent must have an immediate effect at lowest concentrations. At the same time, however, it must also have a lasting effect and only be consumed after a certain period of time (depot effect).

This is especially true of HF.

HF is known as a contact poison. The damage mechanisms are manifold. Its effects are best investigated in skin burns, as these are unfortunately frequent especially in industry.^22^

The damaging mechanisms, such as inhibition of the enzymatic system by HF, its long-lasting penetration into deeper tissue layers, and damage along vascular and neuronal structures seem to be of particular interest in view of the symptoms of retinal toxicity reported after the use of contaminated PFO batches.^23^ The progressive damage, long-lasting corrosiveness, terrible healing potential, and severe necrosis associated with HF burns reveal remarkable parallels to the ocular damage that has been observed.^22^,^23^

The toxic effect of the batches examined and their differences can only be understood when all identified impurities in their different ratios and effects and their interactions are taken into account.

## Conclusion

The Ala^®^octa batches we investigated differ significantly in terms of their individual impurity groups. It should be emphasized that the batches' impurity profiles may change during their shelf life due to reactions among the reactive underfluorinated compounds. Pharmaceutical processing steps involving the impact of energy can trigger such changes. This may be one reason for the significant differences between batches derived from the same raw materials.

The impurity groups we detected can cause different side effects. We identified reactive underfluorinated impurities and their degradation product (HF) as one reason for the toxic effect of Ala^®^octa batches.^3^,^4^ Notably, the individual risks of each type of impurity may be increased by interaction with other impurity groups, as that happens with the supporting effect of surface-modifying compounds during tissue transition.

The time-dependent differences in the cytotoxic effect of the batches tested can be explained by differences in the amount of reactive underfluorinated impurities and varying HF content, as this ratio is unstable over time. Therefore, the detection of both acute and latently toxic impurities of this type, using the so-called H-value as the limit test, plays an important role when characterizing a safe PFO-endotamponade.^4^

This raises the question of whether the current regulations and standards now in force are in fact being adequately and legally enforced. In this context, the current International Organization for Standardization (ISO) standards and Conformité Européenne markings on medical devices in Europe are subject to particular criticism. In these discussions, however, there is often too little distinction drawn between the measures taken to set specific parameters and the steps necessary gaining approval based on fulfilling those parameters (determination of specifications) and for ensuring that the products conform to specifications, that is, that all batches being routinely produced are also in conformity with the specifications and fully comply with approval requirements. The relevant parameters and their determination are set out in the standards of the ISO series. The approval requirements themselves and the requirement for the products' compliance are currently regulated in the Medical Device Direction in Europe. From 2020, the much stricter Medical Device Regulations regulations will apply. One essential regulation is that new risks and findings be identified through continuous market observation, and that they be incorporated within the specifications and standards.

We place this work in this context. There is strong evidence that all detected impurities can be plausibly traced back to the manufacturing of raw materials and their further processing. Applying a risk-based approach, this must be reflected in appropriate specifications with appropriate limits both for the PFO-specific impurities and for those impurities that may contaminate the product through the production of specific dosage forms. Only after separating all impurities, in particular those of reactive underfluorinated impurities, can the batches' stability be achieved and their toxicity (and all other unwanted side reactions) be completely avoided. Our revelation of the different chemical constitutions of individual impurity groups shows that this is only achievable by combining different purification steps that consider the impurities' various chemical properties and are adapted to them.

**Figure 4:**
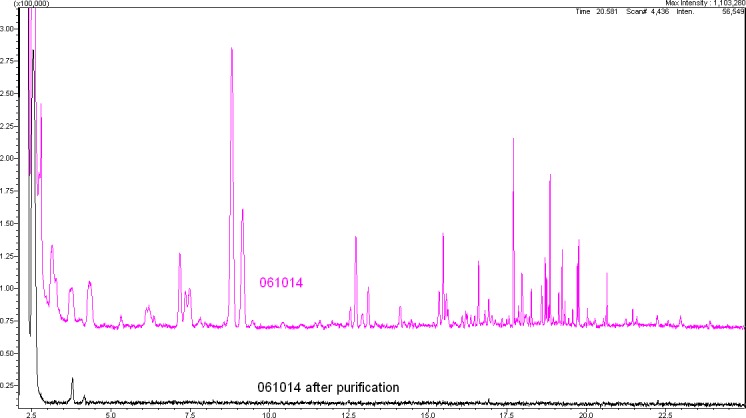
Comparison of the GC/MS results before and after multistage ultra-high-purification of batch 061014.

Figure 4 demonstrates the effectiveness of such a Good manufacturing Practice-controlled purification process using batch 061014 as an example. The toxic and risk-carrying impurities were completely removed, and the previously cytotoxic batch exhibited no more cell-growth inhibition.^4^

The combination of multistage, ultra-high-purification processes and analytical control of their efficiency applying analytical methods specifically optimized for the impurity types can guarantee the safe production of PFCL products.

Definition of the contamination profile and identification of HF as a contaminator possessing extremely high toxic potential can help to develop or adapt treatment strategies to alleviate damage caused by impure PFO endotamponades. The only means of preventing such incidents is, however, the aforementioned combination of carefully monitored manufacturing processes according to GMP and the batch-based testing of the specification adapted to the product-typical contamination profile.

## References

[1] Méndez-Martínez S, Calvo P, Rodriguez-Marco NA, Faus F, Abecia E, Pablo L (2018). Blindness related to presumed retinal toxicity after using perfluorocarbon liquid during vitreoretinal surgery. *Retina Phila Pa*.

[2] Januschowski K, Dimopoulos S, Szurman P (2015). Injection scheme for intravitreal bevacizumab therapy for macular oedema due to central retinal vein occlusion: results of a multicenter study. *Acta Ophthalmol (Copenh)*.

[3] Pastor JC, Coco RM, Fernandez-Bueno I (2017). Acute retinal damage after using a toxic perfluoro-octane for vitreo-retinal surgery. *Retina Phila Pa*.

[4] Menz D-H, Feltgen N, Menz H (2018). How to ward off retinal toxicity of perfluorooctane and other perfluorocarbon liquids?. *Invest Ophthalmol Vis Sci*.

[5] Gross U, Ruediger S, Kolditz L, Reichelt H (1990). Blutersatzstoffe auf Fluorcarbonbasis. *Mitteilungsblatt Chem Ges*.

[6] Meinert H (1994). Perfluorochemicals in ophthalmology: materials and basic principles. *Fluorine in Medicine in the 21st Century: UMIST, Manchester University*.

[7] Sparrow JR, Ortiz R, MacLeish PR, Chang S (1990). Fibroblast behavior at aqueous interfaces with perfluorocarbon, silicone, and fluorosilicone liquids. *Invest Ophthalmol Vis Sci*.

[8] Chang S, Sparrow JR, Iwamoto T, Gershbein A, Ross R, Ortiz R (1991). Experimental studies of tolerance to intravitreal perfluoro-n-octane liquid. *Retina Phila Pa*.

[9] Hoerauf H, Kobuch K, Dresp J, Menz DH (2001). Combined use of partially fluorinated alkanes, perfluorocarbon liquids and silicone oil: an experimental study. *Graefes Arch Clin Exp Ophthalmol*.

[10] Gervits LL (1994). Perfluorocarbon-based blood substitutes: Russian experience. In: Banks RE, Lowe KC, Eds. *Fluorine in Medicine in the 21st Century: UMIST, Manchester University, 18**21 April 1994*. Manchester, UK: Rapra Technology Ltd;.

[11] Meinert H, Roy T (2000). Semifluorinated alkanes A new class of compounds with outstanding properties for use in ophthalmology. *Eur J Ophthalmol*.

[12] Turberg M, Brady J (1988). Semifluorinated hydrocarbons: primitive surfactant molecules. *J Am Chem Soc*.

[13] Kobuch K, Menz IH, Hoerauf H, Dresp JH, Gabel VP (2001). New substances for intraocular tamponades: perfluorocarbon liquids, hydrofluorocarbon liquids and hydrofluorocarbon-oligomers in vitreoretinal surgery. *Graefes Arch Clin Exp Ophthalmol*.

[14] Meinert H, Knoblich A (1993). The use of semifluorinated alkanes in blood-substitutes. *J Int Soc Artif Cells Immobil Biotechnol*.

[15] Menz D, Dresp J (2008). Biocompatibility of highly fluorinated liquids used in ophthalmic surgery. *Fluorine and Health*.

[16] Dresp JH, Menz D-H (2005). Interaction of different ocular endotamponades as a risk factor for silicone oil emulsification. *Retina Phila Pa*.

[17] Dresp JH, Menz D-H (2007). The phenomenon of “sticky” silicone oil. *Graefes Arch Clin Exp Ophthalmol*.

[18] Dresp JH, Menz D-H (2004). Preparation and processing of vitreoretinal instrumentation and equipment as a risk factor for silicone oil emulsification. *Retina Phila Pa*.

[19] Pastor Jimeno JC, de la Rúa ER, Fernández Martínez I, del Nozal Nalda MJ, Jonas JB (2007). Lipophilic substances in intraocular silicone oil. *Am J Ophthalmol*.

[20] International Council for Harmonisation of Technical Requirements for Pharmaceuticals for Human Use (2016). Guideline for Residual Solvents Q3C(R6). *ICH Harmon Guidel*.

[21] Franko J, Meade BJ, Frasch HF, Barbero AM, Anderson SE (2012). Dermal penetration potential of perfluorooctanoic acid (PFOA) in human and mouse skin. *J Toxicol Environ Health A*.

[22] Flusssäure Sander D (1980). Fluoride III-2.3.. http://www/toxcenter.org/stoff-infos/f/fluss-saeure.pdf.

[23] Januschowski K, Irigoyen C, Pastor JC (2018). Retinal toxicity of medical devices used during vitreoretinal surgery: a critical overview. *J Int Ophthalmol*.

